# The rise of photoresponsive protein technologies applications *in vivo*: a spotlight on zebrafish developmental and cell biology

**DOI:** 10.12688/f1000research.10617.1

**Published:** 2017-04-11

**Authors:** Renee Wei-Yan Chow, Julien Vermot

**Affiliations:** 1 Institut de Génétique et de Biologie Moléculaire et Cellulaire, Illkirch, France; 2 Centre National de la Recherche Scientifique UMR8104, Illkirch, France; 3 Institut National de la Santé et de la Recherche Médicale, U964, Illkirch, France; 4 Université de Strasbourg, Illkirch, France

**Keywords:** embryogenesis, CRE, optogenetic technology, fluorescent proteins

## Abstract

The zebrafish ( *Danio rerio*) is a powerful vertebrate model to study cellular and developmental processes *in vivo*. The optical clarity and their amenability to genetic manipulation make zebrafish a model of choice when it comes to applying optical techniques involving genetically encoded photoresponsive protein technologies. In recent years, a number of fluorescent protein and optogenetic technologies have emerged that allow new ways to visualize, quantify, and perturb developmental dynamics. Here, we explain the principles of these new tools and describe some of their representative applications in zebrafish.

## Introduction

Since as early as the 1930s, researchers in classic development and embryology have praised the ease of handling zebrafish embryos and their optical clarity ^1^. These small tropical fishes are easy to house, produce large numbers of externally fertilized eggs (a pair of zebrafish can produce up to 300 fertilized eggs a week), and have relatively short generation times compared with other vertebrate models (around 3 months). They also develop rapidly: within a day of development, the embryonic axes are established, somitogenesis is complete and neural development is under way, and by five days post-fertilization, all major organs have formed and the larvae are able to swim freely and feed. Zebrafish embryos are transparent during the first day of development, allowing direct observation of their embryonic development. Although several types of pigment gradually restrict optical access to deeper structures as the embryos develop, pigmentation can be greatly reduced through the simple addition of chemical drugs (for example, 1-phenyl-2-thiourea, or PTU ^2^) to the water. There are also a large number of pigmentation mutants that survive to adulthood. For example, *nacre* mutants lack skin melanophores ^3^, *sandy* and *albino* mutants show melanin deficiency in both skin and the retinal pigmented epithelium, *roy orbison* and *shady* mutants lack skin iridophores ^4, 5^, and *pfeffer/salz* mutants have greatly reduced numbers of xanthophores ^6^. Combinatorial pigmentation mutants, such as *casper* ^4^, which carry both the *roy orbison* and *nacre* mutations, and *crystal* ^7^, which carry the *nacre*, *roy orbison*, and *albino* mutations, offer optical access to even adult fish without incurring the toxic effects of PTU.

The zebrafish rose to prominence as a model for developmental and cell biology in the mid-1990s, when two large-scale forward genetic screens were carried out ^8^, and a detailed morphological characterization of zebrafish development was performed ^9^. At the time, forward genetics was not technically or economically possible in other prominent vertebrate models, such as frog, chick, and mouse. Although reverse genetics in zebrafish was difficult, microinjection of early zebrafish embryos with either mRNA or antisense morpholino oligonucleotides could be used to transiently overexpress or knockdown gene function ^10, 11^.

By the time the large-scale forward genetic screens for zebrafish were complete, a wave of change had happened in the biological sciences because of improvements in fluorescence microscopy. The optical transparency and small size of the zebrafish embryo could be fully exploited, and zebrafish emerged as a powerful model to image cellular and subcellular developmental events *in vivo*. At first, zebrafish transparency was mainly used to generate fate maps during early embryo development ^12^ and to image neurons of the nervous system ^13^; cells were typically labeled by injection of fluorescent dyes, including fluorescent calcium indicator dyes. The optical transparency of the zebrafish also meant that it was possible to target specific populations of fluorescent neurons for laser ablation ^13^. The development of green fluorescent protein (GFP) and its spectral variants as genetically encoded fluorescent labels made it possible to follow the movements, positions, and interactions of tagged proteins, organelles, and whole cells in a variety of tissues ^14, 15^. That zebrafish transgenic lines expressing genetically encoded fluorescent proteins could be made was first shown in 1995 ^16, 17^. A few years later, a landmark multiphoton time-lapse imaging study found a way to specifically express GFP in the zebrafish vasculature ^18^. This study revealed for the first time the dynamics of angiogenesis in both wild-type and mutant backgrounds and demonstrated the power of fluorescent protein technology when combined with the zebrafish model. When the Tol2 transposon system made zebrafish transgenesis efficient, a large number of transgenic lines expressing fluorescent proteins tagged to different proteins were made, allowing the labeling of various subcellular structures, cells, tissues, and organs ^19^. The Tol2 system was later combined with the Gal4/UAS system to generate a large collection of fish lines that express Gal4 in specific cells, tissues, and organs ^20, 21^—a collection that remains invaluable today.

The mid-2000s heralded the bloom of optogenetics: the use of light to manipulate cell activity using genetically encoded light-responsive proteins. Until this point, the only form of optogenetics was the inactivation of proteins through chromophore-assisted light inactivation, where the chromophore was a fluorescent protein, such as enhanced GFP (EGFP) ^22^. However, our ideas regarding the scope of photoresponsive protein-based tools changed dramatically when a pioneering study showed that, by introducing the gene for channelrhodopsin-2 (a microbial opsin) into cultured mammalian neurons, one could trigger neuronal depolarization using light ^23^. Whereas performing optogenetic experiments on mouse neurons *in vivo* required invasive surgery, delivering light to zebrafish neurons was relatively straightforward and the zebrafish’s small size meant that all neurons from a defined circuit can be monitored at once under a laser scanning microscope. The zebrafish thus played a large part in the “optogenetic revolution” that occurred in the neurosciences, which allowed experimentalists to functionally test the role of identified neurons in specific behaviors for the first time (reviewed in 24– 27). To a limited extent, optogenetic use of membrane-bound opsins that are light-sensitive ion channels, pumps, or G protein-coupled receptors expanded from neuroscience into other fields of biology. Two notable uses of optogenetics in the zebrafish involved the expression of the light-gated pump halorhodopsin and the light-sensitive cation channel channelrhodopsin-2 in zebrafish cardiomyocytes, allowing the study of the formation of the cardiac pacemaker in the developing heart ^28^ and the study of flow propagation in the embryonic vascular network ^29^.

Over the years, the variety of fluorescent proteins has greatly expanded, and they now encompass a broad range of tools that go beyond simple labeling of biological structures. Although optogenetics still is most commonly associated with light-sensitive membrane-bound opsins for the study of neuronal function, recently there has been a proliferation of a new set of optogenetic tools based on light-dependent protein-protein interactions that promise to have broad application to all aspects of developmental biology. In this review, we discuss the advantages and weaknesses of some of the latest developments in fluorescent protein and optogenetic technologies and focus specifically on their use in zebrafish.

## Innovative fluorescent protein technologies

### Color coding cells

One major challenge in developmental biology is to visualize cellular organization in complex tissues; a related challenge is to follow changes in cell morphology and movement over time and to perform lineage tracing and understand the origin of cells and cell populations. Driving fluorescent proteins under certain promoters can restrict fluorescent protein expression to specific cell subpopulations, but the labeling is often too dense to achieve single-cell resolution when one is studying complex tissues, and sometimes there are no available promoters that are specifically expressed in the cells of interest. A decade ago, researchers came up with a cell-labeling technique to distinguish individual neurons in the mouse brain. The technique, called Brainbow, uses stochastic Cre-loxP recombination to express one of several spectrally distinct fluorescent proteins from a single transgene. Since multiple cassettes are integrated at a single genomic site, and the choice within each cassette is made independently, varied combinations of the different fluorescent proteins label individual cells with distinct fluorescent profiles and generate what is effectively a multicolored Golgi stain ^30^. Brainbow constructs were first tested in zebrafish in 2011 ^31^, and several transgenic lines incorporating Brainbow technology, including the Zebrabow series ^32^ and Tg(UAS:brainbow) ^33^, soon followed ( Table 1). The Brainbow strategy has been adopted for cell tracking in several zebrafish tissues to great success. For example, Brainbow technology allowed clonal analysis of the zebrafish heart and revealed how the orchestrated division of cardiomyocytes results in the distinct architectures of the ventricle and atrium ^34, 35^ and how endocardial cell movements contribute to cardiac valve formation ^36^. It also allowed the identification and quantification of collective cell behaviors, enabling zebrafish skin homeostatic maintenance and response to injury ^37^ and the precise manner by which zebrafish retinal ganglion cell axons laminate in the tectum ^33^.

**Table 1.** Brainbow-related zebrafish transgenic lines.

Line	References
Tg(βactin2-Brainbow)	34, 35, 37
Tg(ubi-Zebrabow-S)	32
Tg(ubi-Zebrabow-M)	32
Tg(UAS:Brainbow)	33
Tg(UAS:Zebrabow-V)	32
Tg(UAS:Zebrabow-B)	32
Tg(actb2:multibow)	38

In the original Brainbow design, the expression levels of fluorescent proteins do not follow an even distribution, thus reducing the randomness of fluorescent protein expression and increasing the likelihood that neighboring cells express indistinguishable spectral profiles. Other disadvantages include not only the possibility of changing the color code of the cell because of permanent CRE expression but also the sensitivity of the technique to factors that affect fluorescent protein signal intensity, such as promoter activity, cell depth, or autofluorescence. Recently, there have been efforts to overcome these problems by using independent transgenes for each fluorescent protein, each equipped with a binary ON/OFF switch. In this approach, called Multibow, fluorescent proteins are initially OFF and then probabilistically ON or OFF following Cre recombination ^38^. A zebrafish line generated using this technique takes advantage of our increasing repertoire of fluorescent proteins, expressing no fewer than seven different fluorescent proteins, which have been tagged to three different subcellular localizations (membrane, cytoplasm, and nucleus) to increase variation of labeling ^38^. Although Multibow has not been used for any biological applications yet, it is an interesting new addition to the multicolor labeling toolbox. Given that membrane, cytoplasmic, and nuclear compartments show large variations in fluorescent protein signal intensity, it can be difficult to distinguish the color of one or more of these compartments ^38^. For example, in some cases, the nucleus of the cell is much more brightly labeled than the cytoplasm or membrane, making it difficult to visualize cellular morphology ^38^. Nonetheless, Multibow may prove useful, especially for cell lineage studies where the use of time-lapse imaging is not possible.

A caveat of current multicolor labeling technologies for live embryo imaging is the necessity to image embryos multiple times, including using violet light for the excitation of blue fluorescent proteins, which can cause phototoxicity. This problem is partially elevated by improved microscopy techniques that require fewer excitation photons to achieve the same resolution. A particularly notable milestone in recent years is the adaptation of light-sheet microscopy for the imaging of live embryos ^39, 40^. Light-sheet imaging combines high imaging speed with low light exposure, which, when combined with the optical transparency of zebrafish and standard fluorescent protein labeling techniques, enables many developmental processes to be imaged at unprecedented spatial and temporal resolution. Also significant is the introduction of new point-scanning microscopy setups that apply wavelength mixing to provide simultaneous, efficient, and independent two-photon excitation of more than two spectrally distinct fluorophores, thereby avoiding the use of shorter, more phototoxic wavelengths ^41^. Another deterrent to using Brainbow technology for cell lineage tracing studies is the time lag between when CRE is first delivered/expressed and when fluorescent proteins mature. An alternative strategy that does not have this limitation is to follow optically highlighted cells, as discussed in the section below.

### Highlighting single cells with light

Photoconvertible fluorescent proteins change emission wavelengths in response to irradiation with light of a particular wavelength, whereas photoactivatable fluorescent proteins gain fluorescence after irradiation of light of a particular wavelength ( Table 2). Together, these proteins serve as optical highlighters, allowing the rapid and precise labeling of biological structures using laser light. The first fluorescent proteins advertised as being photoconvertible and photoactivatable were Kaede and photoactivatable GFP (PA-GFP), respectively. Kaede’s photoconvertible properties were discovered serendipitously when the researchers left a vial of the protein overnight by the window and discovered the next day that sunlight had converted the sample from green to red; Kaede is named after the leaves of the Japanese maple that change from green to red in the autumn. Further investigation revealed that the red form of Kaede reflects the protein’s ionized state and that this change in emission wavelength can be effectively and irreversibly induced by illuminating the protein with violet 405-nm light ^42^. By contrast, PA-GFP was created purposely by modifying the structure of GFP through mutagenesis so that the protein would greatly increase its fluorescence after irradiation using violet 405-nm light ^43^. A few years after the introduction of Kaede and PA-GFP, Dronpa, a fluorescent protein that was not only photoactivatable but photoswitchable, was discovered. The fluorescence of this interesting protein can be switched off by blue 488-nm light and switched on again by violet 405-nm light exposure ^44^. Since then, a number of new optical highlighters have been developed, and significant effort has been made to create monomeric versions that perform better in protein fusion studies (reviewed in 45). Optical highlighters have been used in zebrafish to visualize single-cell morphology ^46, 47^; to track cells for cell lineage, migration, or proliferation analyses ^48– 52^; and to mark cells for laser ablation ^53^ ( Table 2).

**Table 2.** Photoconvertible and photoactivatable proteins used in zebrafish.

Protein	Before photomodulation		Photomodulation wavelength, nm	Two-photon activation, nm	After photomodulation		Oligomeric state	Discovery/ Development	Examples in zebrafish
	Excitation, nm	Emission, nm			Excitation, nm	Emission, nm			
Kaede	508	518	405	Inefficient	504	517	Tetramer	42	47, 143
KikGR; mKikGR	507	517	405	Inefficient	583	593	Tetramer; Monomer	144, 145	143, 146, 147
EosFP; mEosFP	506	516	405	Inefficient	571	581	Tetramer; Monomer	148, 149	92, 150, 151
Dendra2	490	507	405	Inefficient	553	573	Monomer	152	50, 51
PSmOrange	548	565	488	Inefficient	636	662	Monomer	58	48
PA-GFP	400	515	405	720–840	504	517	Monomer	43	65
Dronpa	503	518	ON: 405 OFF: 488	780	460	504	Monomer ^a^	44	46

^a^Dronpa in the OFF state exists as monomers. In the ON state, Dronpa forms dimers, which can come together to form tetramers.

**Figure 1.**
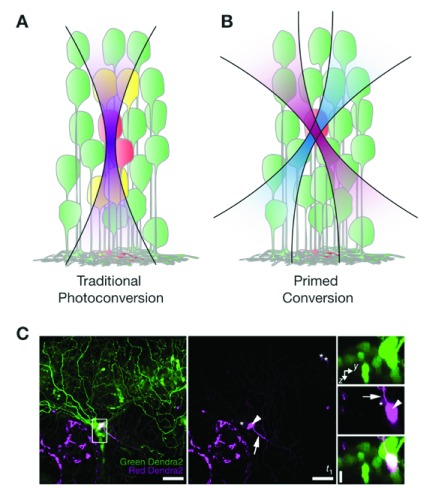
Primed photoconversion. ( **A**) Traditional photoconversion experiments use green-to-red photoconvertible proteins that change from emission after being converted with violet 405-nm light. It is often difficult to specifically photoconvert single cells because the laser beam is not confined in the axial direction. ( **B**) Primed conversion involves first priming green-to-red photoconvertible proteins with blue 488-nm light, before converting the protein with near infra-red 730-nm light. Hence, only cells at the intersection of the two laser beams are photoconverted. ( **C**) Left and middle: Primed photoconversion of Dendra2 in a single neuron in the trigeminal ganglion of a zebrafish at 3 days post-fertilization (maximum-intensity projection of about 82 μm in depth). Right: Higher-magnification axial orientation images of the boxed region in the left frame. Arrowhead indicates the photoconverted cell, and the arrow indicates a neurite extending from the cell body. Asterisks indicate cells that had noticeable signal in the red Dendra2 channel even before photoconversion. Reprinted with permission from Macmillan Publishers Ltd ^56^.

Recently, there have been two exciting developments relating to photoconvertible proteins. The first development is the surprising discovery that the green-to-red photoconvertible protein Dendra2 can be “primed converted”. It turns out that rather than using violet light, one can photoconvert Dendra2 by irradiating the protein first with blue light and then with near-infrared light ^54^. The mechanism responsible for this unusual phenomenon is still unknown, but its usefulness soon became evident. Firstly, infrared light and blue light are less toxic than violet light and penetrate deeper into tissue. Secondly, the dual wavelength mechanism overcomes one of the caveats of green-to-red photoconvertible proteins: they cannot be efficiently photoconverted using multiphoton illumination ^55, 56^. Whereas one-photon illumination is not confined in the axial direction ( Figure 1A), primed conversion ( Figure 1B, 1C) can spatially confine the photoconverted region in all three dimensions by allowing both the priming and the converting beam to meet only at a small axially confined focal volume ^56^. Importantly, primed conversion requires much lower peak power than two-photon photomodulation to accomplish confinement ^56^. The effectiveness of this technique was demonstrated by highlighting neuronal morphology at single-cell resolution in the densely labeled zebrafish brain ^56^. Primed conversion of Dendra2 was also shown to be possible using light from red lasers instead of the more uncommon and expensive near-infrared lasers, albeit at much lower efficiencies ^57^. Given that many transgenic lines expressing Dendra2—including PhOTO zebrafish (photoconvertible optical tracking of zebrafish) lines that ubiquitously label nuclear or plasma membrane proteins using Dendra2 ^51^—are readily available, primed conversion promises to be a useful technique for future studies.

The second development is the long-awaited arrival of new photoconvertible proteins with red-shifted excitation and emission wavelengths, such as PSmOrange and its improved version PSmOrange2 ^58, 59^. These proteins have many advantages over traditional green-to-red fluorescent proteins, not the least of which is that they can be easily used in conjunction with the many available zebrafish lines that already use the green emission wavelength to label biological structures. Another advantage is that the blue-green light needed for one-photon excitation of the protein is less toxic to cells than violet light. One study suggests that PSmOrange2 can also be efficiently photoconverted using infrared light from a two-photon laser ^59^, raising the possibility of spatially confined conversion in tissues using two-photon laser light, although this study was performed in cell culture and the two-photon conversion of PSmOrange2 *in vivo* remains to be verified. Although PSmOrange2 has not been tested in zebrafish, one study reported the injection of H2B-PSmOrange mRNA into one-cell stage zebrafish embryos to track photoconverted cells during ventral habenulae development ^48^. Given the study’s success, we expect PSmOrange and its variants to be much more widely used in the future.

In addition to classic uses of optical highlighters, we expect great applications of single-cell photoconversion and photoactivation in the field of gene expression profiling with the recent development of single-cell genomics. Recent experiments prove its feasibility *in vivo* ^60^ and promise important discoveries in the field of molecular cellular identity and clonal analysis.

### Genetically encoded calcium indicators

Genetically encoded calcium indicators (GECIs) have become essential tools to study calcium signaling in a cell type- or cell compartment-specific manner. Since calcium is a second messenger for neurotransmitter reception and membrane depolarization in neurons, GECIs have been especially useful in the study of neuronal function. Most GECIs fall into one of two main groups: Förster resonance energy transfer (FRET)-based indicators, which are composed of two fluorescent proteins linked by a Ca ^2+^-responsive element, such as the cameleons, and those based on a single circularly permutated fluorescent protein and a Ca ^2+^-responsive element, such as the GCaMPs. Improving the performance of these GECIs has sustained research interest over the last 17 years, and at present the state-of-the-art GCaMP6 series outstrip the performance of the most commonly used synthetic calcium dyes ^61– 63^. Expressed in cultured neurons, the GECIs now possess the sensitivity and kinetics fast enough to detect single action potentials as well as peak fluorescence intensity levels comparable to that of the parental circularly permuted EGFP itself ^61^.

Zebrafish was the first vertebrate model system where GECI expression was used to visualize neuronal activity *in vivo* ^64^. Today, the use of GECIs has been combined with light-sheet microscopy to visualize neuronal activity in the entire zebrafish brain ^7, 65– 69^. Our ability to visualize neuronal activity in whole zebrafish brains has improved dramatically with the arrival of new light-sheet microscope technology, most notably IsoView microscopy ^68^, which generates images of greater spatial resolution by performing fluorescence detection along four orthogonal directions, and two-photon light-sheet microscopy ^69^, which uses light of wavelength greater than that in the visible spectrum to stimulate GECIs and thus has greater tissue penetration and prevents unwanted visually evoked neuronal activity during imaging. Also significant is the introduction of a new zebrafish pigmentation triple mutant, *crystal*, that allows unprecedented optical access to the retina and the brain ^7^.

Beyond studying neuronal function, GECI expression in zebrafish has been instrumental in studying the diverse roles of calcium signaling during development, particularly during cardiovascular development. For example, GECIs were used to identify and analyze stages of cardiac conduction that correspond to cellular and anatomical changes of the developing zebrafish heart and, in combination with a forward genetic screen, to identify conduction-specific mutations ^70^. Recently, GECIs were used to characterize heart conduction defects in a zebrafish model of desminopathy, a type of muscle disorder ^71^, and to show that calcium signaling is critically involved in cilia-mediated blood flow sensing ^72^ and sprouting angiogenesis ^73^ in endothelial cells during early vascular morphogenesis.

Excitingly, the combined improvements in GECI brightness and microscopy have opened up the possibility to study calcium signaling in a cell compartment-specific manner in live zebrafish embryos. For example, SyGCaMP2, a synaptic vesicle calcium reporter that consists of GCaMP2 fused to synaptophysin, has allowed both the identification of synapse localization and the reporting of electrical activity in the zebrafish optic tectum and retina ^74^; migrating zebrafish neutrophils have been shown to display enriched leading-edge calcium flux, a finding in contrast to *ex vivo* studies ^75^; zebrafish myocytes were found to show sustained increase in mitochondrial calcium levels during spontaneous muscle contraction ^76^; and calcium oscillations within cilia were found to be required for asymmetric Nodal signaling in the zebrafish left-right organizer during left-right symmetry breaking ^77^.

Another exciting development is the introduction of red GECIs, which facilitate experiments that require dual-color imaging ^78– 81^. The latest red GECIs—jRCaMP1a,b ^78^, jRGECO1a ^78^, and R-CaMP2 ^79^—approach the performance of GCaMP6. The makers of jRCaMP1a,b and jRGECO1a tested the performance of six red GECIs in zebrafish, and their results suggest that jRGECO1a shows the greatest sensitivity in the animal but that jRCaMP1b shows the fastest on- and off-kinetics ^78^. The authors also advised that jRCaMP1b may be more suited to many optogenetic experiments than jRGECO1a since it does not photoswitch in response to blue light ^78^.

**Figure 2.**
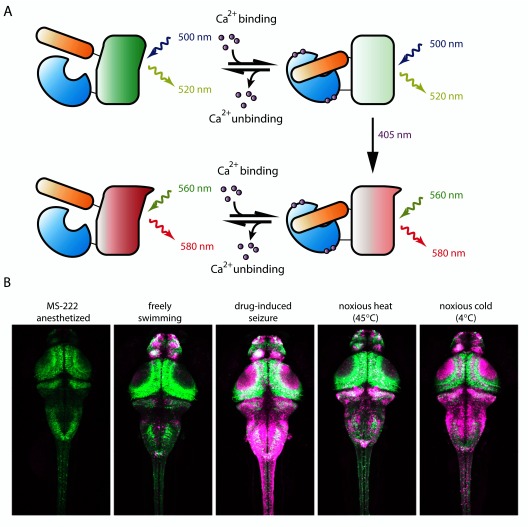
Calcium-modulated photoactivatable ratiometric integrator (CaMPARI). ( **A**) Schematic of CaMPARI function. Both green and red forms of CaMPARI decrease fluorescence in the presence of calcium. CaMPARI is more susceptible to photoconversion by violet 405-nm light when intracellular calcium levels are high and this is most likely due to conformational change of the protein when bound to calcium. ( **B**) Zebrafish larvae after 10 seconds of 405-nm light applied during exposure to different conditions. Each image represents a maximum-intensity Z-projection of CaMPARI signal in a confocal stack through a 4- to 5-days post-fertilization larval zebrafish brain expressing CaMPARI pan-neuronally under the elavl3 promoter. Reprinted with permission from the American Association for the Advancement of Science ^86^.

With the advancement in photoactivatable and photoconvertible protein technology (see earlier section), photoactivatable and photoconvertible GECIs are now also available, allowing optical selection of cells/cell compartments for calcium imaging ^82– 85^. A particularly interesting photoconvertible GECI, called CaMPARI (calcium-modulated photoactivatable ratiometric integrator), undergoes efficient green-to-red photoconversion by violet 405-nm light when calcium levels are high ^86^ ( Figure 2A). Although both red and green forms of CaMPARI respond dynamically to calcium in a manner similar to other GECIs, its uniqueness lies in its ability to retroactively report calcium activity over defined periods of time. For example, by illuminating brains expressing CaMPARI for a few hours with violet 405-nm light, neurons that experience higher levels of activity become red fluorescent while neurons with low levels of activity remain green fluorescent. The permanent labeling alleviates the need to image the right cells at the right time to observe neuronal activity. The expression of CaMPARI in zebrafish has allowed the visualization of integrated neuronal activity in zebrafish subjected to different conditions—anesthetized, swimming freely, treated with seizure-inducing compounds, or put in extreme heat or cold ( Figure 2B)—and the technique promises a number of potential uses, ranging from functional connectomics to transcriptional profiling based on cellular calcium signaling levels.

### Protein timers

Traditional molecular biology and biochemistry have provided us with the identity of key proteins and regulatory networks involved in many developmental processes. However, the spatiotemporal dynamics of their regulation remains poorly understood, and imaging the proteome *in vivo* remains a significant challenge. Methods that have been used to monitor the behavior of proteins in zebrafish embryos include fluorescence recovery after photobleaching (FRAP) and its related technologies (fluorescence loss in photobleaching, fluorescence loss after photoactivation, and fluorescence localization after photobleaching) ^87– 93^ and fluorescence correlation spectroscopy ^91, 94– 98^. Recently, a new method has been developed to image protein turnover, based on the use of tandem fluorescent protein timers (tFTs) that change their emission wavelengths as the protein ages ^99^.

Using fluorescent proteins that change emission wavelength as the protein ages itself is not a new idea. However, previous versions of fluorescent protein timers consist of a single fluorescent protein that switches color over time as its fluorophore undergoes successive chemical reactions ^100– 102^. These protein timers have low brightness and a tendency to form oligomers, which perturbs the behavior of the tagged proteins, and to our knowledge have not been used for *in vivo* studies. In this context, Knop *et al*. created a new type of fluorescent protein timer, the tFT, which involves a tandem fusion of a fast maturing protein of one color and a slower maturing protein of another color ^103^. The idea is that by measuring the ratio of one color to the other, one could track protein movement within the cell; by using mathematical models that incorporate the known maturation kinetics of the fluorescent proteins, one could infer the protein turnover rate constants.

The first study featuring tFTs fused superfolder GFP (sfGFP), which reaches its peak intensity very quickly, with mCherry, which matures more slowly and reaches its peak intensity a few hours later. This tFT was then used to uncover the stable nature and asymmetric inheritance of nuclear pore complexes and identify regulators of N-end rule-mediated protein degradation in yeast ^103^. tFTs were introduced to the zebrafish model via two elegant studies investigating the dynamics of chemokines and cell-cell adhesion in the zebrafish lateral line primordium. In the first study, the authors tagged Cxcr4b, the receptor of chemokine Cxc112a, with sfGFP and the slower-maturing fluorescent protein TagRFP. The red/green fluorescence ratio at the plasma membrane was used as an indicator of the age of receptor populations, and the rate of ligand-triggered internalization was used to measure Cxc112a chemokine levels. By doing so, the authors effectively showed that a group of cells migrating collectively can self-generate gradients of chemokine activity across its length via polarized receptor-mediated internalization ^104^. This study has established that tFTs can be sensitive, reliable tools for visualizing protein dynamics over many hours in live zebrafish embryos. Application of protein timers is broad, and other than the zebrafish line primordium ^104– 106^, protein timers have been used to study protein relocalization and stabilization during cardiac development ^107^ and somite morphogenesis ^108^.

It must be emphasized that tFTs are not neutral tags, and careful selection of fluorophores for use in tFTs has to be tailored to the expected turnover kinetics of the endogenous protein of interest. Given the wide range of fluorescent proteins with different spectral properties, and different maturation and degradation kinetics available, choosing the best combination of fluorescent proteins may be daunting. Recently, a study compared the performance of different GFPs within tFTs in yeast and provided comprehensive advice on the effects of the stability of the GFP fold on tFT behavior ^109^. Mathematical models of timer kinetics have been developed and were verified by applying a set of timers to once again investigate chemokine signaling gradients across the zebrafish posterior lateral line primordium. Interestingly, one of their findings was that FRET between the fast- and slow-maturing fluorophores increases timer signal, a point that may be useful for future timer designs. An interactive web application based on the models, called TimerQuant, has been made freely available to guide experimentalists on which fluorescent proteins to choose, to interpret timer readouts, and to detect differences in protein half-life ^110^.

The relative ease of use of tFTs compared with fluorescence correlation spectroscopy and FRAP-based methods—including the ability to capture temporal information in a single snapshot, the possibility to perform experiments with relatively simple microscopy setups, and the availability of open source software—will likely mean that tFTs will become much more widely used in the future.

### Protease activity reporters

Proteases are enzymes that perform hydrolysis of peptide bonds and are important in many biological and developmental processes through their ability to activate or inactivate their target proteins. Biosensors for monitoring protease activity typically involve a peptide specific for the protease of interest flanked by a pair of fluorescent proteins that undergo FRET. When the peptide is cleaved, the two fluorescent proteins separate and the FRET signal disappears. However, the signal of FRET reporters is weak because of small fluorescence change in the donor and acceptor fluorophores and the use of these reporters *in vivo* is limited.

Recently, Shu *et al*. introduced ZipGFP, a GFP-based protease reporter that achieves about 10-fold fluorescence increase upon caspase activation ^111^. To create ZipGFP, the researchers exploited the fact that GFP can be split into two fragments that have sufficient affinity to self-reassemble and form the complete, fluorescent β-barrel protein ^112^: the two parts of GFP were each flanked with heterodimerizing coiled coils, which prevent the two parts from self-assembling; the consensus protease cleavage sequence for caspase-3 was incorporated into the peptides linking the heterodimerizing coiled coils and the GFP fragments, such that when cleaved by caspase-3, the two parts become uncaged and free to bind. The researchers injected mRNA encoding ZipGFP into live zebrafish to visualize caspase activity and apoptosis of the vertebrate *in vivo*. They found that the spatial pattern of ZipGFP fluorescence at 2 days post fertilization was consistent with a previous study based on terminal deoxynucleotidyl transferase dUTP nick end labeling (TUNEL) staining and further showed that apoptosis first occurred at the rostral part of the forebrain before appearing in other parts of the brain and the trunk, a finding that would have been extremely difficult to observe with the limited spatial resolution of TUNEL staining using fixed samples. The design of ZipGFP will likely be used to create reporters of many other proteases, allowing us to study protease dynamics with unprecedented spatiotemporal resolution.

## Emerging optogenetic technologies

The investigation of dynamic developmental processes requires fast and flexible methods to perturb protein function. The classic method to perturb protein function in zebrafish embryos is simply to add drugs to the swimming media. Since zebrafish embryos are small, many small molecules are able to penetrate even deep tissues. However, drugs delivered this way diffuse to all tissues, not only to cells and tissues of interest. With the advance of zebrafish genetics, proteins can be more finely regulated both spatially and temporally at the transcription level. Tissue-level spatial resolution of expression or recombination is often achieved by the use of cell type-specific promoters, whereas temporal control is achieved via the administration of small-molecule compounds, such as in the LexPR ^113^, TetON, or TetOFF systems ^114^, or activation of heat shock promoters ^115, 116^. Photoactivatable morpholinos and photo-cleavable morpholinos can also be used to switch on and off morpholino activity, respectively, using ultraviolet (UV) 365-nm light ^72, 90, 117, 118^. However, morpholinos are typically injected into embryos at the one-cell stage and delivered to all cells in the developing embryo, and as such UV irradiation of a specific region of the embryo activates photomorpholinos in all cells of the region, not just in cells of interest. Importantly, the temporal control afforded by photomorpholinos can be applied once only; once you activate a photomorpholino with UV light, you cannot control its deactivation. Recently, novel optogenetic tools based on light-induced protein-protein interactions offered the opportunity to control protein function reversibly down to the subcellular scale, not only at the transcription level but at all stages of a protein’s life.

**Figure 3.**
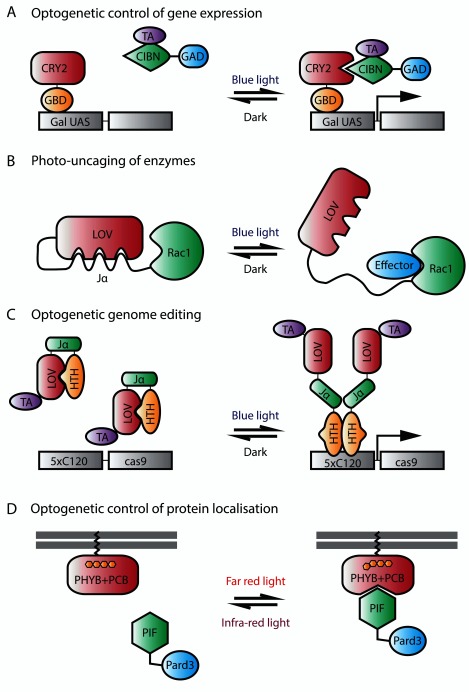
Optogenetic approaches in zebrafish. ( **A**) Optogenetic control of gene expression. In this approach, cryptochrome2 (CRY2) is fused to the Gal4 DNA-binding domain, GBD, and CIBN is fused to a transcription activator (TA) and to GAD, a domain required for transcriptional activation through interaction with transcriptional machinery. In the dark, CRY2 does not associate with CIBN. Upon illumination with blue light, CRY2 binds to CIBN, leading to the expression of the gene of interest. ( **B**) Photo-uncaging of enzymes. This photoactivatable Rac1 consists of a light-oxygen-voltage (LOV) domain connected to a Jα helix, which is then fused to Rac1. In the dark, LOV binds to Jα and sterically inhibits the interaction of Rac1 with its downstream effectors. Upon blue light illumination, the helix unwinds and frees Rac1, thus restoring its activity. ( **C**) Optogenetic genome editing. Here, the LOV domain is fused to a TA and to a helix-turn-helix (HTH) DNA-binding domain via the Jα linker. In the dark, the LOV domain interacts with HTH. Upon illumination with blue light, HTH is released and allowed to dimerize and bind to DNA, leading to the expression of *cas9*. ( **D**) Optogenetic control of protein localization. ** In this system, phytochrome B (PHYB) is tethered to the membrane via the CAAX motif while PIF is fused to Pard3. In the dark, PHYB does not associate with PIF. Upon illumination with far-red light, PHYB undergoes a conformational change, allowing it to bind to PIF, thus recruiting Pard3 to the membrane. The conformational change of PHYB, and hence its interaction with PIF, is reversible upon infra-red light illumination. This system requires the external cofactor phycocyanobilin (PCB).

Proteins that interact only with other proteins or protein domains under light conditions are common in organisms that photosynthesize, such as plants, algae, and cyanobacteria ( Table 3). The three most well-known photosensitive proteins that have been used for controlling other proteins are cryptochrome2 (CRY2), the light-oxygen-voltage (LOV) domain, and phytochrome B (PhyB). Of these, the most commonly used photosensitive protein is CRY2, which binds to the basic-helix-loop-helix (bHLH) transcription factor CIB1 upon blue light illumination. In cultured cells, association occurs on a subsecond timescale and reversion is a spontaneous process with half-life times occurring within minutes ^119^. The CRY2 mutation E490G can also act as an optogenetic system where blue light stimulation induces oligomerization of the protein ^120^. The feasibility of creating optogenetic gene switches in zebrafish using the CRY2/CIB1 system was recently confirmed in a proof-of-principle study ^121^ ( Figure 3A), although biological applications of the system in zebrafish are still pending.

**Table 3.** Light-inducible protein interaction systems used in zebrafish.

Photosensitive protein	Mechanism	Light, nm	Activation/Inactivation time in cell culture	Use in zebrafish	References
CRY2	CRY2/CIB1 heterodimerization	488	Seconds/Minutes	121	119
LOV domain: AsLOV2	Unfolding of Jα helix	465	Seconds/Seconds	127	122, 153
LOV domain: TAEL-HTH	TAEL-HTH homodimerization	465	Seconds/Seconds ^a^	126	124
LOV domain: AUREO1-LOV	Homodimerization	465	Seconds/Seconds	128	154
PhyB	PhyB/PIF6 heterodimerization	633/750	Milliseconds/Milliseconds (inactivation with far-red light)	129, 130	155, 156

^a^Based on parent protein EL222.

The second most used system is the LOV domain, which forms a covalent bond with the flavin cofactor and undergoes a conformational change upon blue light stimulation. As with CRY2, the conformational change is spontaneously reversible in the dark. Since its discovery, various LOV domain-derived optogenetic modules have been developed ^122– 126^. Some of these are based on binding the LOV domain directly to an effector protein and rely on light to uncage the effector protein. Others are based on LOV domain heterodimerization with natural or engineered binding partners in response to light. Still others are based on the ability of LOV domains to homodimerize. A LOV-based Rac1 photo-uncaging system ( Figure 3B) has been used in the zebrafish to study neutrophil motility ^127^. The study successfully demonstrated that localized activation of the small GTPase at the leading edge of neutrophils is sufficient to direct their migration with precise temporal and spatial control and that PI(3)K-mediated regulation of neutrophil motility occurred through both the modulation of Rac-mediated protrusion at the leading edge and anteroposterior polarity of F-actin dynamics ^127^. In another study, photoactivatable Nodal receptors based on LOV domain homodimerization were used to study cell fate specification during zebrafish gastrulation ^128^. Through these photoactivatable Nodal receptors, the study found that extending Nodal signaling within the zebrafish embryonic organizer induces prechordal plate and suppresses endoderm specification. Most recently, a light-inducible gene expression system based on EL222, a naturally occurring light-responsive transcription factor that contains a LOV domain, was optimized for low toxicity in zebrafish ^126^. In the dark, the LOV domain binds to a helix-turn-helix (HTH) DNA-binding domain. Upon irradiation with blue light, the interaction between the LOV domain and the HTH is disrupted, and HTH is free to homodimerize and bind to a regulatory element termed C120. The authors were able to use the system, named TAEL, to induce ectopic endodermal cells in the presumptive ectoderm via targeted induction of the transcription factor *sox32* and modulation of Nodal signaling dynamics by inducing *lefty1* expression. Furthermore, the authors demonstrated how their LOV-based system can be used together with the latest genome-editing technology, CRISPR/Cas9, to induce gene mutations specifically in cells irradiated by light ^126^ ( Figure 3C).

Lastly, PhyB binds to its partner PIF3/6, a transcription factor with a helix-loop-helix structure, upon red light stimulation and dissociates with PIF3/6 upon exposure to far-red light. Advantages of this system over CRY2/CIB1 and LOV-based systems include the lower toxicity and deeper penetration of red/far-red light compared with blue light and the greater control of protein activity afforded by the ability to control the OFF state as well as the ON state of the interaction with light. The PhyB/(PIF3/6) system has not been widely used, because it requires a chromophore, phycocyanobilin (PCB), which is not naturally present in many multicellular organisms, including zebrafish. The PhyB/PIF3 system can be applied to the superficial cells of the zebrafish simply by adding PCB to the medium, as has been done by 129 to control the nuclear localization of proteins with red light. A more recent study ^130^ was able to apply this system to even deep tissues of the zebrafish by injecting a purified version of PCB into one cell of the embryo at the 16-cell stage along with a truncated version of PhyB that optimizes its expression ( Figure 3D). The authors generated a Pard3-PIF6 fusion construct and were able to direct the apical polarity protein Pard3 and recruit its binding partner Pard6 to specific membrane locations in the neural progenitors using red laser light.

**Figure 4.**
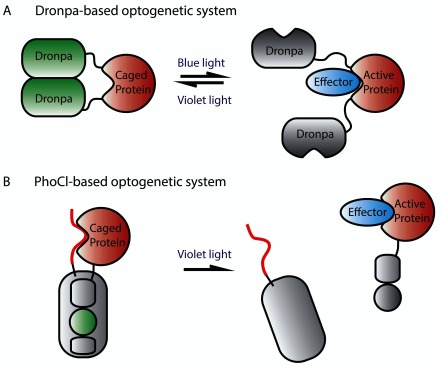
Fluorescent protein-based optogenetic systems. ( **A**) The protein of interest is fused to two Dronpa proteins. Initially, Dronpa is in its “ON” fluorescent state and forms dimers that cause inhibition of protein function by steric interference. Upon irradiation with blue light, the Dronpa dimers dissociate and release protein activity and lose fluorescence. Dronpa can be triggered to form dimers and fluoresce again by irradiation with violet light. ( **B**) The protein of interest is fused to PhoCl, as is a linker that blocks the protein’s function. Initially, the protein of interest is caged and PhoCl fluoresces green light. Irradiation with violet light irreversibly cleaves PhoCl, releasing the protein of interest, and the chromophore’s green fluorescence is lost.

A promising new area in the optogenetics field is the modification of fluorescent proteins for use as optogenetic regulators of protein function, in part because they have the potential advantage of being sensors of their own activity. So far, two fluorescent proteins that have been modified for this purpose are Dronpa, a photoswitchable fluorescent protein, and mMaple, a photoconvertible fluorescent protein. As discussed previously, the fluorescence of Dronpa can be switched off by blue 488-nm light and switched on again by violet 405-nm light exposure. Another property of this protein is that light can be used to change its oligomerization status. In its fluorescent state, Dronpa monomers bind to one another to form dimers and then these dimers come together to form tetramers. Switching off Dronpa fluorescence using blue light converts the protein to monomers, whereas switching on Dronpa fluorescence using violet light reverts the protein to its oligomerized state. Taking advantage of this property, an engineered variant of Dronpa containing a point mutation, which makes the protein less likely to form tetramers in low micromolar concentrations and facilitates off-photoswitching, has been used as an optogenetic module that allows reversible uncaging of the effector protein ^131^ ( Figure 4A). To our knowledge, the use of Dronpa for photocaging has not been implemented in zebrafish or other *in vivo* systems.

The second fluorescent protein that has been used for optogenetics, mMaple, converts from green to red fluorescence upon violet light (about 400-nm) irradiation. Recently, this protein was modified such that violet light irradiation causes irreversible cleavage of the protein as well as loss of green fluorescence ^132^ ( Figure 4B). This modified version of mMaple, called PhoCl (photocleavable protein), can be used to cage proteins in an inactive state until release by photocleavage. The use of high-energy violet light to elicit photocleavage can be circumvented by using primed conversion with lower-energy 458-nm and 730-nm light. Importantly, unlike other optogenetic systems, where constant illumination is required to sustain protein activity, once PhoCl is cleaved, uncaged proteins persist until they are degraded by normal cellular processes. Also, PhoCl systems have much lower levels of dark-state activity than systems based on photosensitive proteins whose photo-induced conformational change is reversible. PhoCl systems are thus highly advantageous in scenarios where low levels of protein activity before light irradiation and prolonged protein activity after light irradiation are desired. The inventors of PhoCl demonstrated its versatility by using the protein to engineer photoactivatable Cre-recombinase, photoactivatable Gal4 transcription factor, and a photoactivatable viral protease. The system has yet to be tested *in vivo* but, should it work in zebrafish, its unique properties will undoubtedly be valuable to the zebrafish community.

As can be seen from the examples above, optogenetic strategies can be applied to control cell activities as wide ranging as differentiation, migration, and polarization. The versatility of photo-inducible protein-protein interaction systems is such that optogenetics can be used to study almost all areas of developmental and cell biology. That both blue light systems (CRY2/CIBN, LOV) and red/far-red light systems (PHYB/PIF) are workable in zebrafish means that both systems could potentially be used simultaneously. A downside of current zebrafish-tested optogenetic systems is that they are slightly “leaky”, in that some photosensitive protein modules expressed in cells exist in the activated state even in the dark. As in the case with fluorescent proteins, the tool kit of photo-inducible protein-protein interaction systems is also expected to expand from the discovery and isolation of new naturally occurring photosensitive proteins from different organisms. These systems will respond to different wavelengths of light and will have different activation/inactivation kinetics as well as different changes in binding affinity between the protein dimers during light and dark states, making them suitable for different applications.

## Conclusions and outlook

We hope that the examples we have provided illustrate how the latest advances in fluorescent protein and optogenetic technologies can be applied to answer important developmental questions in living zebrafish embryos and larvae. Since data obtained from *in vitro* studies do not always match up to what happens *in vivo*, zebrafish constitute an increasingly valuable model to study vertebrate biology permitting the powerful use of phototechnologies. The advances described here come at a time when microscopy tools and approaches ^133– 138^, as well as computational tools for data management and image analysis, are also undergoing rapid development (for some computational tools specific for zebrafish, see 139– 142). With the increasing trend of creating microscopes that are optimized for simultaneous optogenetic manipulation and high resolution, long-term imaging remains one of the major goals in the near future. Exciting areas of development will be analyzing and extracting meaningful information from gene expression profiling combined with quantitative information about the cell behaviors and identities *in vivo*. There is a lot to bet that continual interactions between chemists, biologists, computational scientists, engineers, and physicists will be key to reaching that level of understanding.
